# Diffuse Normolipemic Plane Xanthoma Associated With Monoclonal Gammopathy

**DOI:** 10.7759/cureus.89071

**Published:** 2025-07-30

**Authors:** Catarina Cerqueira, Joana Sobreiro Silva, Carlos Moreira Nogueira, Miguel Ribeiro, Ana Paula Vieira

**Affiliations:** 1 Department of Dermatology and Venereology, Unidade Local de Saúde de Braga, Braga, PRT; 2 Department of Surgical Pathology, Unidade Local de Saúde de Braga, Braga, PRT

**Keywords:** mgus, monoclonal gammopathy, non-langerhans histiocytosis, plane xanthoma, xanthomatosis

## Abstract

Diffuse normolipidemic plane xanthoma is a rare acquired dermatosis of the non-Langerhans histiocytosis subtype, often associated with underlying hematologic disorders, with half of the reported cases linked to lymphoproliferative disorders. This case report describes a 52-year-old woman presenting with asymptomatic, yellowish plaques symmetrically distributed on the eyelids, cervical region, axillae, upper back, and upper thighs, which had evolved over a two-year period (images of the eyelids and upper back are not provided). Skin biopsy was consistent with the diagnosis of plane xanthoma. Laboratory evaluation showed normal lipid levels, but elevated serum IgG and the presence of a monoclonal band, leading to the diagnosis of monoclonal gammopathy of undetermined significance (MGUS) after further workup. Over 18 months after diagnosis, the patient remains under follow-up in both the dermatology and hematology departments, with no signs of disease progression.

## Introduction

Xanthomas are a common manifestation of lipid metabolism disorders. They include hyperlipemic xanthoma, normolipidemic xanthoma, and necrobiotic xanthogranuloma [1]. Diffuse normolipidemic plane xanthoma (DNPX) is a rare acquired dermatosis of the non-Langerhans histiocytosis subtype, described by Altman and Winkelmann [2-5]. The disease is slightly more common in men than women and typically manifests in the sixth or seventh decade of life, regardless of serum lipid levels [6]. Clinically, it presents as symmetrical, flat, yellow patches or mildly raised, yellow-orange plaques that develop gradually, most commonly affecting the eyelids, neck, upper trunk, and flexural areas [2-5]. Histologically, it is characterized by the presence of foam cells (macrophages that have engulfed lipid droplets), along with varying numbers of Touton giant cells, lymphocytes, and foamy histiocytes [4]. Data about DNPX are generally scarce, primarily limited to individual case reports, with fewer than 110 cases documented in the medical literature [6].

This article was previously presented as an oral communication at Reunião do Verão 2024 on June 28, 2024.

## Case presentation

**Figure 1:**
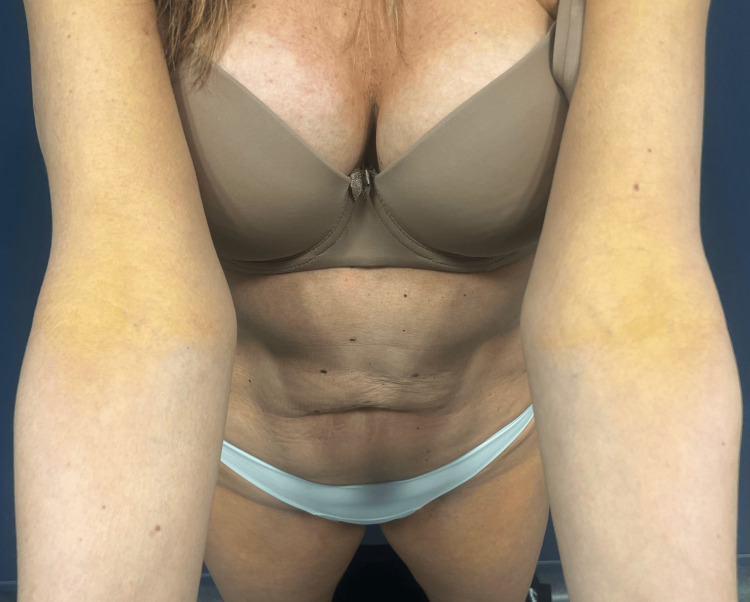
Bilateral yellowish plaques on the antecubital fossae

**Figure 2:**
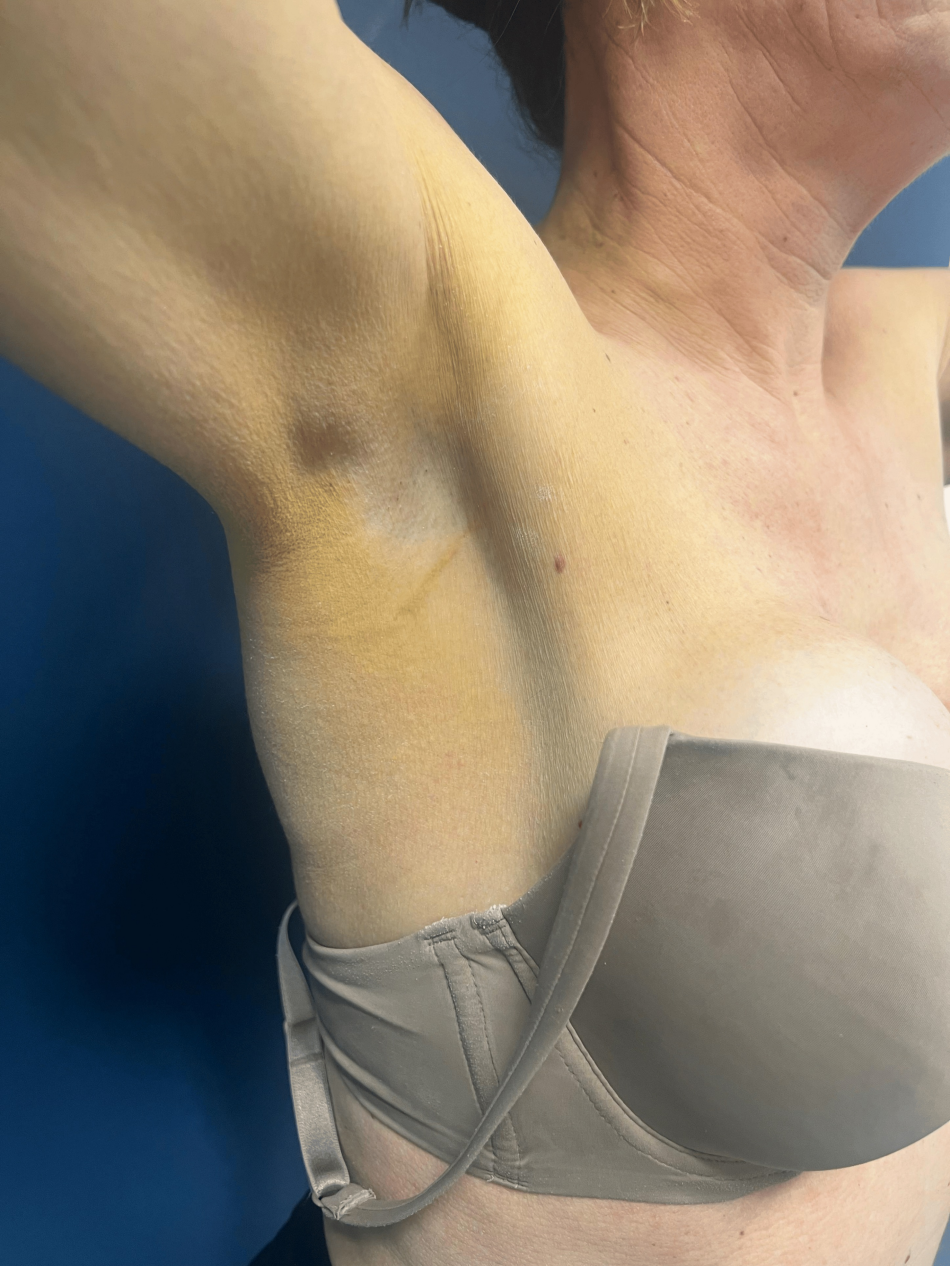
Yellowish plaque on the right axilla

**Figure 3:**
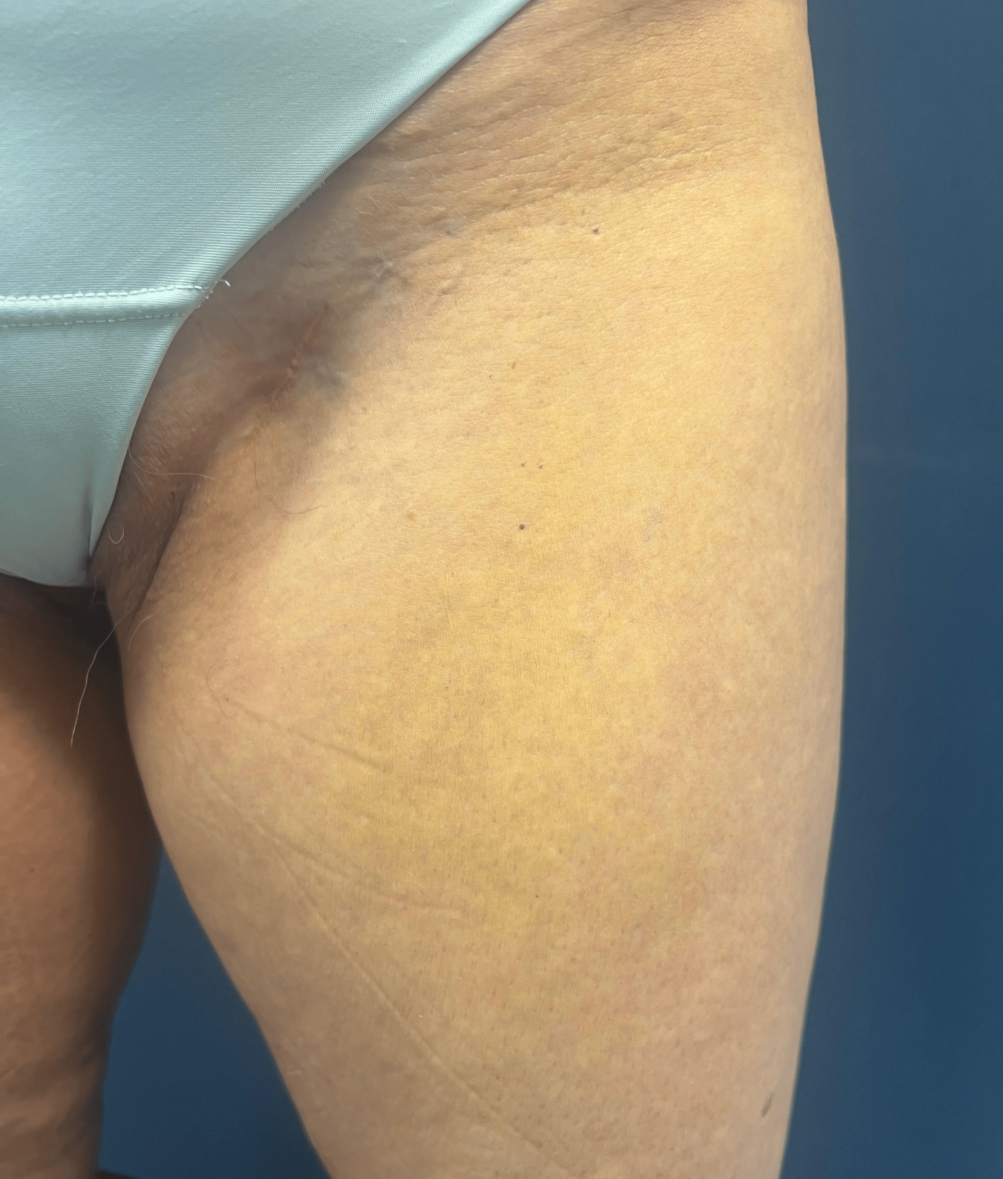
Yellowish plaque on the left thigh

We report the case of a healthy 52-year-old woman referred to the dermatology department by her primary care physician due to the progressive emergence of asymptomatic, yellowish plaques with a symmetrical distribution on the eyelids, cervical region, axillae, upper back, antecubital fossae, and upper thighs over the past two years (eyelids and upper back images not provided) (Figures 1-3).

**Figure 4:**
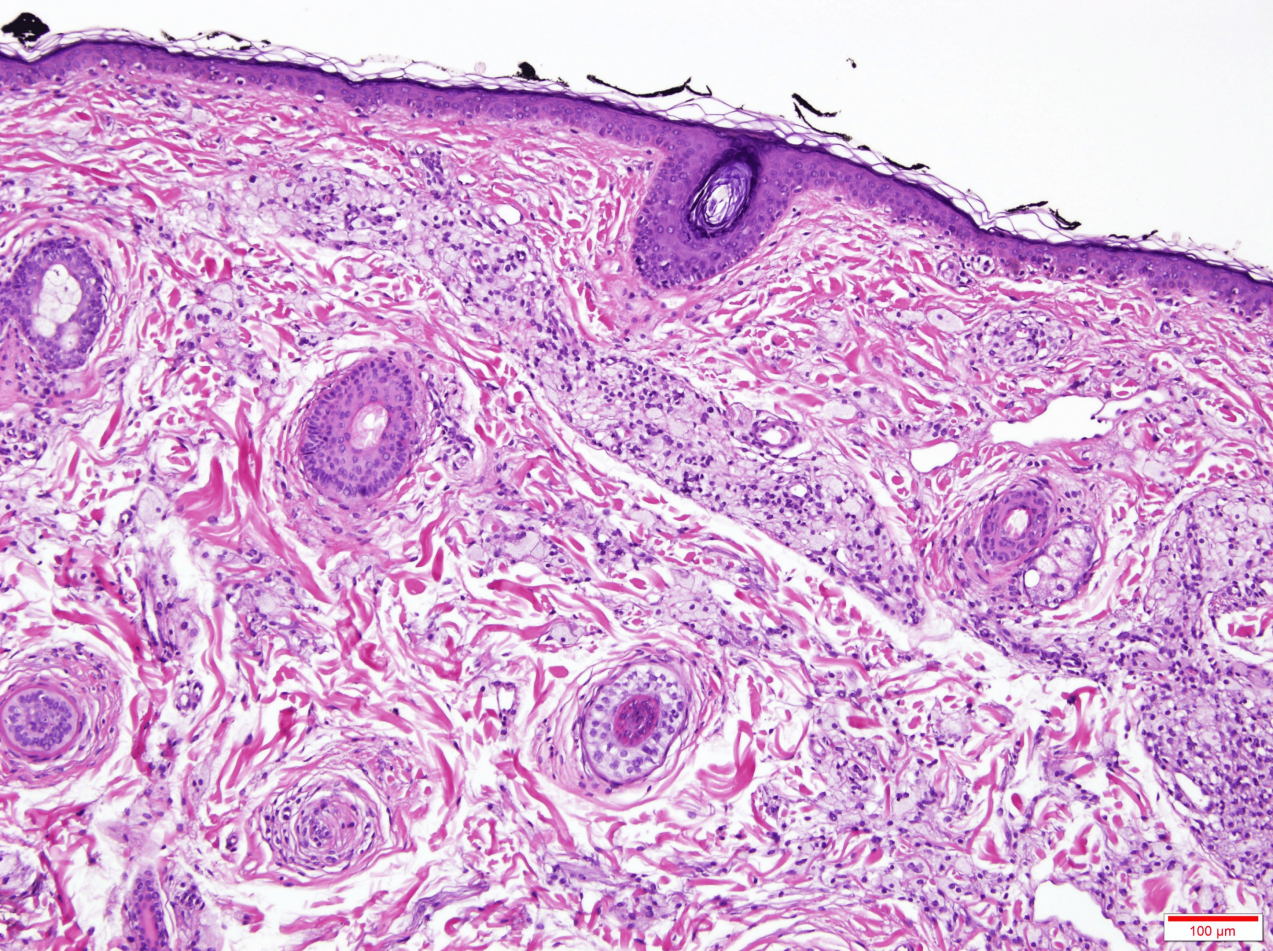
Scattered foamy histiocytes within the reticular dermis (H&E, 100×) H&E: hematoxylin and eosin

No other body areas were affected, and no additional skin lesions were noted. The patient denied any recent infections, trauma, or initiation of new medications. A 6-mm punch biopsy from the left axilla revealed scattered foamy histiocytes within the reticular dermis, consistent with a diagnosis of plane xanthoma (Figure 4).

A comprehensive laboratory study, including complete blood count, metabolic panel, and fasting lipid profile, was all within normal limits. However, serum protein electrophoresis demonstrated an elevated IgG level of 29.43 g/L, with the presence of an M-band in the gamma region.

Additional investigation included a bone marrow aspirate and whole-body positron-emission tomography (PET) scan. The bone marrow aspirate showed 1% plasma cells, 81% of which were clonal kappa cells, consistent with monoclonal gammopathy of undetermined significance (MGUS). The PET scan showed no abnormalities.

Over 18 months after the diagnosis, the patient remains under follow-up in both dermatology and hematology departments, showing no signs of disease progression, and has opted against any form of cosmetic treatment.

## Discussion

Diffuse normolipemic plane xanthoma (DNPX) can be classified into two subtypes: idiopathic and disease-associated. More than half of reported DNPX cases are linked to lymphoproliferative disorders [3]. DNPX has been linked to a range of hematologic disorders, most prominently multiple myeloma, monoclonal gammopathies (including MGUS), chronic granulocytic and lymphocytic leukemias, Waldenström’s macroglobulinemia, cryoglobulinemia, and various forms of lymphoma [2-5,7,8]. DNPX may precede the diagnosis of systemic conditions by years, necessitating regular clinical and laboratory monitoring for disease progression.

Although the exact pathogenesis remains unclear, in gammopathy-associated DNPX, it is hypothesized that monoclonal IgG binds to circulating low-density lipoprotein, forming immune complexes that are more readily phagocytosed by macrophages [3].

The prognosis of DNPX is contingent on the underlying disorder, and treatment should focus accordingly. Improvement in cutaneous lesions has been reported following remission of the associated hematological disease, with recurrence of skin findings potentially signaling relapse [9]. Although DNPX itself is typically not associated with significant systemic morbidity, the cutaneous lesions may be cosmetically distressing [4]. A range of therapeutic options is available for managing this condition. In cases of localized involvement, surgical excision of individual lesions may be performed. Additional treatment modalities include chemabrasion, dermabrasion, and ablative laser therapies, notably those employing the erbium-doped yttrium aluminum garnet laser [5].

## Conclusions

DNPX is a rare and often asymptomatic dermatological condition that may signal underlying systemic diseases, particularly monoclonal gammopathies. In this case, the diagnosis of DNPX was pivotal in uncovering an associated MGUS, emphasizing the importance of comprehensive evaluation for underlying conditions in such presentations. While systemic therapies are not routinely indicated in asymptomatic cases without disease progression, cosmetic interventions should be offered for disfiguring lesions.

## References

[1] Szalat R, Arnulf B, Karlin L (2011). Pathogenesis and treatment of xanthomatosis associated with monoclonal gammopathy. Blood.

[2] Altman J, Winkelmann RK (1962). Diffuse normolipemic plane xanthoma. Generalized xanthelasma. Arch Dermatol.

[3] Stockman A, Delanghe J, Geerts ML, Naeyaert JM (2002). Diffuse plane normolipaemic xanthomatosis in a patient with chronic lymphatic leukaemia and monoclonal gammopathy. Dermatology.

[4] Cohen YK, Elpern DJ (2015). Diffuse normolipemic plane xanthoma associated with monoclonal gammopathy. Dermatol Pract Concept.

[5] Mendes SR, Gameiro AR, Coutinho I, Cardoso JC (2021). Diffuse normolipaemic plane xanthoma in a patient with monoclonal gammopathy. BMJ Case Rep.

[6] Baykal C, Erdem Y, Çiftçi FK, Çevik AA, Öztürk Sarı Ş, Büyükbabani N (2024). Diffuse normolipemic plane xanthoma: remarkable dermatological findings observed in a series of patients. Balkan Med J.

[7] Kim KJ, Lee DP, Suh HS, Lee MW, Choi JH, Moon KC, Koh JK (2004). Diffuse plane xanthoma in a patient with chronic myeloid leukemia. J Dermatol.

[8] Kyle RA, Therneau TM, Rajkumar SV (2006). Prevalence of monoclonal gammopathy of undetermined significance. N Engl J Med.

[9] Falk L, Dyall-Smith D, Stolz W, Coras-Stepanek B (2019). Diffuse plane xanthoma developing in association with prior monoclonal gammopathy. BMJ Case Rep.

